# Color Normalization in Breast Cancer Immunohistochemistry Images Based on Sparse Stain Separation and Self-Sparse Fuzzy Clustering

**DOI:** 10.3390/diagnostics15182316

**Published:** 2025-09-12

**Authors:** Attasuntorn Traisuwan, Somchai Limsiroratana, Pornchai Phukpattaranont, Phiraphat Sutthimat, Pichaya Tandayya

**Affiliations:** 1 Department of Computer Engineering, Faculty of Engineering, Prince of Songkla University, Karnjanavanich Rd., Songkhla 90110, Thailand; 6710130010@psu.ac.th (A.T.); somchai.l@psu.ac.th (S.L.); 2 Department of Electrical Engineering, Faculty of Engineering, Prince of Songkla University, Karnjanavanich Rd., Songkhla 90110, Thailand; pornchai.p@psu.ac.th; 3 Department of Mathematics, Faculty of Science, Kasetsart University, Bangkok 10900, Thailand

**Keywords:** breast cancer, color deconvolution, histopathological images, immunohistochemistry, fuzzy clustering

## Abstract

**Background and Objective**: The color normalization of breast cancer immunohistochemistry (IHC)-stained images helps change the color distribution of undesirable IHC-stained images to be more interpretable for the pathologists. This will affect the Allred score that the pathologists use to estimate the drug quantity for treating breast cancer patients. **Methods**: A new color normalization technique based on sparse stain separation and self-sparse fuzzy clustering is proposed. **Results**: The quaternion structural similarity was used to measure the quality of the normalization algorithm. Our technique has a structural similarity score lower than other techniques, and the color distribution similarity is closer to the target. We applied automated and unsupervised nuclei classification with Automatic Color Deconvolution (ACD) to test the color features extracted from normalized images. **Conclusions**: The classification result from our unsupervised nuclei classification with ACD is similar to other normalization methods, but it offers an easier perception to the pathologists.

## 1. Introduction

Breast cancer is prevalently diagnosed among women, particularly those under 40, and is a significant cause of mortality, accounting for approximately 44,800 deaths annually in this age group [1]. Pathologists play a crucial role in clinical care by diagnosing breast cancer, determining tumor malignancy, assessing its growth within the breast, and identifying any spread to lymph nodes or other organs. This evaluation typically involves examining stained cancer tissues under a light microscope.

A major issue in evaluating histopathological images, particularly for scoring, arises from color variations caused by differences in stain operator protocols, exposure times, and slide scanner specifications. These inconsistencies significantly impact the quality of feature extraction from the images. To address this, color normalization techniques are employed to standardize image colors, making them more general and consistent.

Historically, image normalization can be achieved by adjusting the colors of a source image to match a target image, which is a process that can be performed using image editing software like GNU Image Manipulation Program (GIMP) or Adobe Photoshop. More advanced methods include histogram-matching algorithms [2], which were initially developed for grayscale images but are adaptable for color images by matching individual color channels. Another approach, the color-matching algorithm, specifically adjusts the mean and standard deviation of lαβ channels.

Macenko et al. put forth a normalization algorithm for histological slides in 2009, which employed color deconvolution for the identification of stain components, subsequently utilizing singular value decomposition (SVD) projection for normalization [3]. Although this approach demonstrated efficacy, it failed to maintain the structural integrity of the tissue, thereby impacting diagnostic outcomes. The guidelines by the College of American Pathologists (CAP) emphasize the necessity of accurate histological imaging to ensure reliable diagnoses. The Laboratory General Checklist [4] established by the CAP necessitates the maintenance of the structural integrity of tissue to avert misdiagnosis, thereby reflecting an ethical obligation to patients. The World Health Organization (WHO) asserts that the diagnosis of tumors emphasizes the significance of the preservation of the tissue structure [5]. In response to this issue, Vahadane et al. refined the color deconvolution technique to safeguard the tissue structure, designating their approach as structure-preserving color normalization [6]. More recently, advanced deep learning models such as StainGAN were employed for unpaired image-to-image translation to facilitate the transfer of stylistic elements in digital histological images [7]. Additionally, fuzzy clustering methodologies were proposed to mitigate uncertainty in the analysis of histological images and enhance color normalization.

The ambiguity inherent in histological image analysis constitutes one of the principal challenges. Maji and Mahapatra proposed the application of circular clustering within the fuzzy approximation domain for the purpose of color normalization of histological images [8]. They employed the round-fuzzy circular cluster model to generate values in the weighted hue histograms of both the source and template images prior to the implementation of non-negative matrix factorization, which was aimed at achieving effective stain separation. Furthermore, comprehensive probability modeling or the Bayesian methodology was utilized to ascertain stain separation in histological images [9,10].

Most existing normalization methods primarily focus on hematoxylin and eosin (H&E) stained slides due to the greater availability of open datasets for H&E compared to immunohistochemistry (IHC) staining. H&E staining provides basic morphological information but lacks molecular details like antigen expression, which IHC staining offers. IHC staining is crucial for pathologists to predict cancer cell growth, and its evaluation involves counting different types of nuclear staining and cell populations. Given the large volume of slides pathologists process, automated image analysis systems were developed for biomarker scoring [11,12,13,14,15]. Unnormalized IHC images can lead to incorrect cell labeling, potentially resulting in inappropriate treatments and affecting cancer cell growth. Furthermore, empirical evidence demonstrated a statistically significant enhancement in diagnostic confidence upon applying medical image normalization [16].

This paper introduces a color normalization method specifically for IHC-stained images. It adapts techniques previously used for H&E stained images, despite the difference in the number of perceived colors (two in H&E vs. three in IHC), by finding a better structure-preserved normalization method to prepare IHC images. The subsequent sections of this manuscript are described as follows. The materials and methodologies are presented in Section 2. Section 3 presents a comparative analysis of the qualitative and quantitative outcomes derived from various benchmark algorithms against our proposed methodology. The discourse is articulated in Section 4. Finally, Section 5 encapsulates the research conducted within this paper.

## 2. Materials and Methods

### 2.1. Database Description

In the present study, our immunohistochemical (IHC) images were obtained from the Department of Pathology at the Faculty of Medicine, Prince of Songkla University. These images were procured from four general and regional hospitals situated in the southernmost provinces of Thailand between January and June 2022. The total number of cases encompassed 151, which were sourced from Naradhiwas Rajanagarindra Hospital, Pattani Hospital, Yala Regional Hospital, and Sungaikolok Hospital. They were identified with ductal carcinoma in situ (DCIS) or epithelial breast cancer at the ages of 23–79 years. Of the 151 participants included, 91 (60.3%) were below 50 years of age, and 60 (39.7%) were above 50 years of age. Furthermore, 131 (86.8%) had invasive ductal carcinoma, 6 (4%) had DCIS, and 14 (9.3%) had pathological types of cancer. The research protocol received approval from the Human Research Ethics Committee of Naradhiwas Rajanagarindra Hospital (REC 001/2564). Furthermore, this investigation conformed to the principles delineated in the Declaration of Helsinki.

The breast tissue images were acquired utilizing a light microscope (Eclipse 80i advanced research microscope, Nikon Instech Co., Ltd., Tokyo, Japan) at a magnification of 40×. These images were stored in a 24-bit color JPEG format. The resolution of the images is 720 × 900 pixels. A singular image may encompass two distinct types of nuclei: cancerous nuclei and non-cancerous nuclei. Each nucleus is stained utilizing two distinct stain colors: blue (immunonegative stain) and brown (immunopositive stain). Pathologists concentrate on quantifying the number of immunopositive nuclei in conjunction with the count of immunonegative nuclei. Five images exhibiting various variations were randomly selected for inclusion in this study.

### 2.2. Color Deconvolution (CD)

Color deconvolution constitutes a sophisticated image analysis technique formulated to delineate and quantify immunohistochemical staining. Its principal aim is to establish a versatile and robust methodology for the objective immunohistochemical assessment of samples subjected to up to three distinct stains, including horseradish peroxidase staining developed with 3,3′ diaminobenzidine (DAB), hematoxylin, and eosin. This methodological approach aspires to address the challenges posed by conventional color transformation techniques by precisely isolating the contributions of individual stains, even in scenarios where they co-localize or exhibit overlapping absorption spectra. The intensities of light transmitted through a specimen (A) can be characterized using the Beer–Lambert law [17]. The correlation between the intensity of light traversing the specimen $\left(I_{C}\right)$ and the intensity of light entering the specimen $\left(I_{0,c}\right)$, in conjunction with the absorption factor (c), is delineated as follows [11]:

(1) $$I_{C}=I_{0,C}\exp\left(-Ac_{C}\right).$$

The subscript *C* denotes the specific detection channel. The concentration of the stain exhibits a non-linear relationship with the RGB color values [3]. Consequently, the RGB color values are not suitable for the purposes of separation and quantification of the concentration of the stain. The optical density (OD) can be articulated as shown below:

(2) $$OD_{C}=-log\left(\frac{I_{C}}{I_{0,C}}\right)=Ac_{C}.$$

Color deconvolution [11] constitutes a methodological approach aimed at the identification of the stain vector $\left(V\in R^{m\times r}\right)$ and the absorption factor $\left(S\in R^{r\times n}\right)$ through the process of decomposing $OD_{R,G,B}\in R^{m\times n}$, where m=3 for the RGB channels, *n* is the number of pixels, and *r* is the number of stains. The intensities of light that are transmitted through a specimen $\left(OD_{R,G,B}\right)$ may be expressed as a matrix representation of *V*, while *S* can be articulated as the matrix denoting the saturation levels of each individual stain as shown below:

(3) $$OD_{R,G,B}=VS.$$

Furthermore, the procedural framework for converting the RGB color space ($I_{R,G,B}$) into the optical density (OD) domain [18] or the Beer–Lambert transformation (BLT) yields the following result:

(4) $$OD_{R,G,B}=-log\left(\frac{I_{R,G,B}}{I_{{\left(R,G,B\right)}_{\mathrm{blank}}}}\right),$$

where $I_{{\left(R,G,B\right)}_{\mathrm{blank}}}$ is the illuminating light intensity on the sample (usually 255 for 8-bit images).

Conversely, the procedure for converting the OD color space into the RGB color space, or the inverted Beer–Lambert transformation (IBLT), is articulated as follows:

(5) $$I_{R,G,B}=I_{{\left(R,G,B\right)}_{\mathrm{blank}}}exp\left(-OD_{R,G,B}\right).$$

### 2.3. Contrast Stretching (CS)

The Spatio-Temporal Retinex-like Envelope with Stochastic Sampling (STRESS) algorithm [19], conceptualized by Kolås et al., is engineered to emulate the adaptive functions of the Human Visual System. Its fundamental purpose is to compute local reference black and white points for each chromatic channel contained within an image. This computation involves the estimation of two envelope functions—maximum ($E_{\max}$) and minimum ($E_{\min}$)—which encapsulate the image signal and exhibit gradual variation. These envelopes are distinguished by their adherence to the signal, exhibiting smoothness, edge preservation, and convergence to the global maximum for $E_{\max}$ and global minimum for $E_{\min}$. The algorithm derives these envelopes for each pixel through the application of a random spray model. Upon the determination of the envelopes, the value of each pixel is modified to enhance contrast, highlight details, and equilibrate the three color channels, thereby effectively executing color correction. It facilitates local contrast enhancement, automatic color adjustment, and high dynamic range image rendering. Each pixel in the image $\left(p_{0}\right)$ undergoes an update as shown below:

(6) $$p_{\mathrm{stress}}=\frac{p_{0}-E_{\min}}{E_{\max}-E_{\min}},$$

(7) $$E_{\min}=p_{0}-\bar{v}\bar{r},$$

and

(8) $$E_{\max}=p_{0}+\left(1-\bar{v}\right)\bar{r}=E_{\min}+\bar{r}.$$

The variables $\bar{r}$ and $\bar{v}$ present within the equations denote the average of the sample range and the average of the relative pixel values, which are computed as shown below:

(9) $$\bar{r}=\frac{1}{N}\sum_{i=1}^{N}r_{i},$$

(10) $$\bar{v}=\frac{1}{N}\sum_{i=1}^{N}v_{i},$$

where *N* represents the number of iterations, $r_{i}$ is the range of the samples, and $v_{i}$ is the relative value of the center pixel given as shown below:

(11) $$r_{i}=s_{i}^{\max}-s_{i}^{\min},$$

(12) $$v_{i}=\left\{\begin{array}{cc} \frac{1}{2}, & \mathrm{if}\;r_{i}=0\\ \frac{p_{0}-s_{i}^{\min}}{r_{i}}, & \mathrm{otherwise} \end{array}\right..$$

In this context, $s_{i}^{\max}$ and $s_{i}^{\min}$ denote the uppermost and lowermost sample values, respectively, derived from a spray that is ascertained through a selection of stochastic samples originating from a disk of radius R centered at the point $p_{0}$, which is articulated as follows:

(13) $$s_{i}^{\max}=\max_{\left\{0,\ldots ,M\right\}}p_{j},$$

(14) $$s_{i}^{\min}=\min_{\left\{0,\ldots ,M\right\}}p_{j},$$

when the number of samples is denoted as *M*, and the random sample values are denoted as $p_{j}$. In the context of color imagery, the computation is executed independently for each individual color channel.

Finally, *R* should equilibrate detail retention and illumination rectification. For instance, in dimly lit images, an augmented *R* may more accurately gauge global illumination, whereas in high-contrast scenes, a diminished *R* circumvents excessive smoothing. The algorithm may employ multi-scale methodologies, administering disparate *R* values. The selection of *M* and *N* should consider the image’s noise level and intricacy. Noisy images may necessitate larger *M* and *N* to average out noise influences. An enhanced *R* typically mandates larger *M* and *N* to guarantee adequate sampling density within the disk, thus avoiding sparse or erroneous envelope estimates.

### 2.4. Stain Separation (SS)

Macenko et al. introduced a computational framework that systematically detects the appropriate stain vectors corresponding to an image [3], thus facilitating color deconvolution and normalization processes. The objective of this methodology is to reconcile histological slides subjected to disparate processing conditions into a unified, standardized framework, which consequently enhances both quantitative analytical capabilities and visual uniformity. In order to ascertain the optimal stain vector (*v*), it is necessary to utilize solely β. The algorithm is delineated as in Algorithm 1.
**Algorithm 1** Automatic Color Deconvolution Algorithm [3]
     **Input:** RGB Slide, β
1:**procedure** FindOptimalStainVectors2:    Convert RGB to OD using Equation (4)3:    Remove data with OD intensity less than β4:    Calculate SVD on the OD tuples5:    Create a plane from the SVD directions corresponding to the two largest singular values6:    Project data onto the plane and normalize them into a unit length7:    Calculate the angle of each point with respect to the first SVD direction8:    Find robust extreme values back to the OD space
     **Output:** Optimal Stain Vectors


Furthermore, non-negative matrix factorization (NMF) may be employed for the purpose of stain separation [20]. Given that the stain is capable of absorbing light but is unable to emit it, therefore, the stain vector (*V*) and the absorption factor (*S*) as articulated in Equation (3) must be non-negative. As a result, it is feasible to determine *V* and *S* by resolving the following equation:

(15) $$\min_{V,S}\frac{1}{2}{∥OD_{R,G,B}-VS∥}_{F}^{2},\;\mathrm{such}\;\mathrm{that}\;V,S\geq 0.$$

Vahadane et al. [6] introduced a new cost function that enhances Equation (15) through the incorporation of *l*1 sparsity regularization applied to the stain vector (*V*), where each stain has been indexed by *j* as shown below:

(16) $$\begin{array}{cc} \; & \min_{V,S}\frac{1}{2}∥OD_{R,G,B}{-VS∥}_{F}^{2}+\lambda \sum_{j=1}^{r}{∥S\left(j,:\right)∥}_{1}, \end{array}$$

such that $V,S\geq {0,∥V\left(:j\right)∥}_{2}^{2}=1$. λ is the parameter for sparsity and regularization. Establishing λ=0 diminishes SNMF to NMF. The optimal value can be ascertained through a grid search by identifying the minimum error of the projected stain color matrix or the correlation of the projected stain density maps [6].

Additionally, sparse coding methodologies [21] may be employed to infer *S*, while dictionary learning techniques [22] are applicable for the estimation of *V*. Consequently, Vahadane et al. utilized the SPArse Modeling Software version 2.6 (SPAMS) [23] for the purpose of estimating these parameters.

### 2.5. Fuzzy Clustering (FC)

The Robust Self-Sparse Fuzzy Clustering Algorithm (RSSFCA) represents an innovative methodology formulated for the purpose of image segmentation [24], explicitly targeting two prevalent challenges encountered in conventional fuzzy clustering algorithms: heightened sensitivity to outliers attributable to non-sparse fuzzy memberships and excessive image segmentation resulting from an insufficiency of local spatial information. RSSFCA offers two significant contributions: initially, it incorporates a regularization framework under the Gaussian metric into the objective function of fuzzy clustering algorithms to attain fuzzy memberships characterized by sparsity, thereby diminishing noise and enhancing clustering efficacy. Furthermore, it presents a connected-component filtering mechanism predicated on an area density balance strategy to address the issue of image over-segmentation, which is comparatively simpler and more rapid than the integration of local spatial information for the elimination of minor areas. Empirical findings demonstrate that RSSFCA proficiently alleviates the sensitivity to outliers and the problem of over-segmentation, thereby producing superior image segmentation results comparing to previous leading algorithms in the field. In order to effectively address sparse fuzzy memberships, a regularization term $\gamma \sum_{i=1}^{c}\sum_{j=1}^{n}u_{ij}^{2}$ was delineated within the objective function as follows:

(17) $$\tilde{J}=\sum_{i=1}^{c}\sum_{j=1}^{n}u_{ij}\Phi \left(x_{j}|v_{i},\Sigma_{i}\right)+\gamma \sum_{i=1}^{c}\sum_{j=1}^{n}u_{ij}^{2},$$

where $x_{j}$ represents an instance of unlabeled data, $v_{i}$ denotes the corresponding centroid of the cluster, and $u_{ij}$ signifies the membership degree of $x_{j}$ relative to the clustering center $v_{i}$, which was constrained such that $0\leq u_{ij}\leq 1$ and $\sum_{i}^{c}u_{ij}=1$ across *c* distinct clusters. Furthermore, $\Phi (x_{j}|v_{i},\Sigma_{i})$ is delineated as shown below:

(18) $$\Phi \left(x_{j}|v_{i},\Sigma_{i}\right)=\ln\left(-\rho \left(x_{j}|v_{i},\Sigma_{i}\right)\right),$$

where $\rho (x_{j}|v_{i},\Sigma_{i})$ denotes the Gaussian probability density function represented as shown below:

(19) $$\rho \left(x_{j}|v_{i},\Sigma_{i}\right)=\frac{exp\left(-\frac{1}{2}{\left(x_{j}-v_{i}\right)}^{T}\right)\Sigma_{i}^{-1}\left(x_{j}-v_{i}\right)}{{\left(2\pi \right)}^{\left(D/2\right)}{\left|\Sigma_{i}\right|}^{\left(1/2\right)}}.$$

In this context, *D* represents the dimensionality of the input data, while $\sum_{i}$ denotes the covariance matrix that encapsulates the intra-class variability of the *i*th class. A reduction in Σ results in a negative value of Φ, which subsequently induces significant inaccuracies in distance calculations and resultant misclassification. Consequently, the issue was addressed by employing $\Phi^{\prime }\left(x_{j}|v_{i},\Sigma_{i}\right)$ in place of $\Phi (x_{j}|v_{i},\Sigma_{i})$ as shown below:

(20) $$\Phi^{\prime }\left(x_{j}|v_{i},\Sigma_{i}\right)=\left\{\begin{array}{cc} \Phi -min(\Phi ), & \mathrm{if}\;min(\Phi )<0\\ \Phi , & \mathrm{otherwise} \end{array}\right..$$

Consequentially, their final objective function is defined as shown below:

(21) $${\tilde{J}}^{\prime }=\sum_{i=1}^{c}\sum_{j=1}^{n}u_{ij}\Phi^{\prime }\left(x_{j}|v_{i},\Sigma_{i}\right)+\gamma \sum_{i=1}^{c}\sum_{j=1}^{n}u_{ij}^{2}.$$

Moreover, ${\tilde{J}}_{j}^{\prime }$ can be separated into *c* sub-problems as shown below:

(22) $${\tilde{J}}_{j}^{\prime }=min\sum_{i=1}^{c}\left(u_{ij}\Phi^{\prime }\left(x_{j}|v_{i},\Sigma_{i}\right)+\gamma u_{ij}^{2}\right).$$

The update $v_{i}$ can be calculated by solving $\frac{\partial {\tilde{J}}_{j}^{\prime }}{\partial v_{i}}=0$ as shown below:

(23) $$v_{i}=\frac{\sum_{j=1}^{n}u_{ij}x_{j}}{\sum_{j=1}^{n}u_{ij}}.$$

Furthermore, the update $\Sigma_{i}$ from $\frac{\partial {\tilde{J}}_{j}^{\prime }}{\partial \Sigma_{i}}=0$ can be calculated as shown below:

(24) $$\Sigma_{i}=\frac{\sum_{j=1}^{n}u_{ij}{\left(x_{j}-v_{i}\right)}^{T}\left(x_{j}-v_{i}\right)}{\sum_{j=1}^{n}u_{ij}}.$$

The comprehensive outline of their methodology is delineated in Algorithm 2, wherein the inputs consist of the number of clusters (*c*), the regularization parameter (γ), the convergence threshold (η), and the maximum number of iterations (*T*). Finally, the algorithm’s outputs are denoted as $\tilde{U},\tilde{V},\tilde{\Sigma }$, which are instrumental for the segmentation of pixels within the image.
**Algorithm 2** RSSFCA Algorithm [24]
     **Input:**
*c*, γ, η, *T*
1:**procedure** RSSFCA2:    Initialize the membership $U^{\left(0\right)}$ and the clustering centers $V^{\left(0\right)}$ using the FCM algorithm3:    Initialize the covariance matrix $\Sigma^{\left(0\right)}$ utilizing the membership and clustering centroids derived from step 2 and Equation (24), and augment it with an identity matrix (I)4:    t←15:    Update $U^{\left(t\right)},V^{\left(t\right)},\Sigma^{\left(t\right)}$ using Equations (22)–(24)6:    Update the objective function ${\tilde{J}}^{\prime (t)}$ using Equation (17)7:    **if** $max|{\tilde{J}}^{\prime (t)}-{\tilde{J}}^{\prime (t-1)}\leq \eta |$ or t≥T **then**8:        Stop9:    **else**10:        t←t+111:        Go to Step 4
     **Output:** $\tilde{U},\tilde{V},\tilde{\Sigma }$


### 2.6. Structure-Preserving Color Normalization (SPCN)

Structure-Preserving Color Normalization (SPCN) is a technique designed to standardize the color appearance of histological images while preserving their underlying biological structure [6]. This method is built upon sparse non-negative matrix factorization (SNMF) for stain separation, which decomposes images into sparse and non-negative stain density maps. SPCN works by replacing the color basis of a source image with that of a pathologist-preferred target image while maintaining the source’s original stain concentrations. This ensures that the structural information, captured in the stain density maps, remains intact, and only the color appearance is altered. This approach addresses the issue of color variations in histological images caused by differences in staining protocols, raw materials, and scanner responses, making images more comparable for analysis by pathologists and software.

In order to achieve a normalized color representation from a source image (*s*) to a target image (*t*), it is imperative to decompose the source optical density ($OD_{s}$) into the product of the matrices $V_{s}$ and $S_{s}$, while concurrently decomposing the target optical density ($OD_{t}$) into the matrices $V_{t}$ and $S_{t}$, as delineated in Equation (16). Subsequently, the matrix $S_{s}$ necessitates normalization according to the following formulation:

(25) $$S_{s}^{norm}\left(j,:\right)=\frac{S_{s}\left(j,:\right)}{S_{s}^{RM}\left(j,:\right)}S_{t}^{RM}\left(j,:\right),j=1,\ldots ,r,$$

where $S_{i}^{RM}=RM\left(S_{i}\right)\in R^{rx1},i=\left(s,t\right)$ and RM(•) denotes the pseudo-maximum value of each row vector at the 99% threshold. Finally, the normalized source will be computed as shown below:

(26) $$OD_{s}^{norm}=V_{t}S_{s}^{norm}.$$

### 2.7. Quaternion Structural Similarity (QSSIM)

Quaternion Structural SIMilarity (QSSIM) is a visual quality matrix (VQM) designed for color images, representing a vectorial expansion of the traditional Structural SIMilarity (SSIM) index using Quaternion Image Processing (QIP) [25]. Unlike scalar methods that often fail to adequately measure combined degradations like blur and desaturation, QSSIM employs a true vectorial approach, treating each color pixel as a single quaternion number. This allows QSSIM to measure changes in both luminance and chrominance vectors simultaneously, making it particularly effective in predicting the visual quality of color images subjected to complex degradations, such as those caused by the color crosstalk effect. Existing VQMs primarily focus on either luminance or chrominance changes, offering superior correlation with human subjective tests for combined degradations.

QSSIM possesses the capability to evaluate the simultaneous degradation attributed to both blurriness and desaturation. The formulation presented in Equation (27) encompasses components of luminance ($\mu_{q}$), chromatic components ($\sigma_{q_{q}}$), and the cross-correlation of color ($\sigma_{q}$), each of which is delineated as follows:

(27) $$QSSIM_{ref,deg}=\left|\left(\frac{2\mu_{q_{\mathrm{ref}}}\cdot \mu_{q_{\deg}}}{\mu_{q_{\mathrm{ref}}}^{2}+\mu_{q_{\deg}}^{2}}\right)\left(\frac{2\sigma_{q_{\mathrm{ref},\deg}}}{\sigma_{q_{\mathrm{ref}}}^{2}+\sigma_{q_{\deg}}^{2}}\right)\right|,$$

where the standard deviations of the source (ref) and processed images (deg) are represented by $\sigma_{q_{\mathrm{ref}}}$ and $\sigma_{q_{\deg}}$, respectively.

Moreover, the first term in QSSIM is a luminance comparison term that measures the similarity in average brightness (mean intensity) between two images or image patches. Luminance reflects the overall illumination level, which is critical for visual perception. The second term is a structural term. It measures the correlation of pixel intensity patterns, capturing structural similarity (e.g., edges, textures). A high measurement indicates that the processed image preserves the structural details of the source image.

### 2.8. Classification of Nuclei in Breast Cancer IHC Based on Automatic Color Deconvolution (CNACD)

The classification of breast cancer nuclei in immunohistochemical (IHC) images can be conducted approximately utilizing a solitary pixel representing the nucleus. The process of stain separation must be implemented on the pixel through the application of Algorithm 1. Subsequently, each RGB stain value is consolidated into a singular grayscale value to facilitate enhanced comparative analysis. The maximum stain value will serve as the criterion for determining the classification of the nuclei. This methodology is encapsulated in Algorithm 3.
**Algorithm 3** CNACD  Algorithm
     **Input:** RGB Pixels
1:**procedure** PredictPixels2:    V←FindOptimalStainVectors(Input,∞)3:    $S\leftarrow \mathrm{LARS}\text{-}\mathrm{LASSO}(X={\left(OD_{Input}\right)}^{T},D=V,\lambda_{1}=0.1)$4:    $S_{R,G,B}\leftarrow \mathrm{ConvertODtoRGB}\left(S\right)$5:    $S_{pos},S_{neg}\leftarrow \mathrm{SeperateStain}\left(S_{R,G,B}\right)$6:    $S_{pos}\leftarrow \mathrm{ConvertRGBtoGRAY}\left(S_{pos}\right)$7:    $S_{neg}\leftarrow \mathrm{ConvertRGBtoGRAY}\left(S_{neg}\right)$8:    Result←newArray9:    **for** i←0toSizeOf(Input) **do**10:        **if** $S_{pos}\left[i\right]>S_{neg}\left[i\right]$ **then**11:           Result.push(−)12:        **else**13:           Result.push(+)14:    Output←Result
     **Output:** Predicted Results


### 2.9. The Proposed Normalization Method

**Figure 1:**
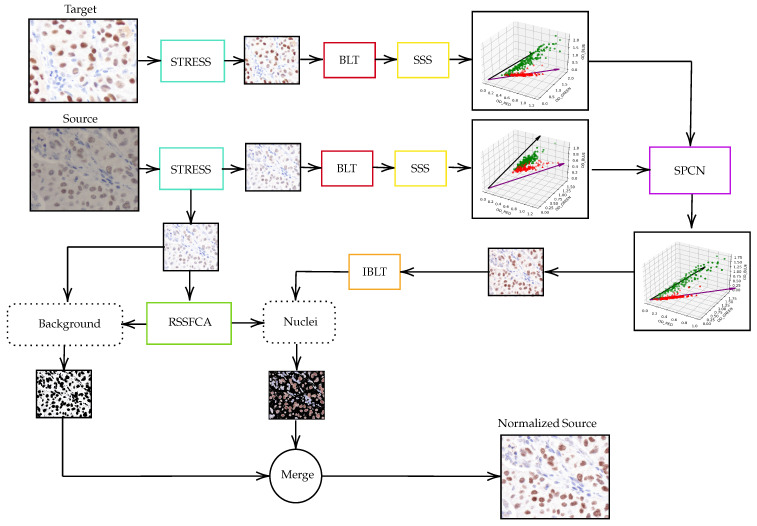
Schematic representation of our proposed methodology for color normalization in immunohistochemistry (IHC) images.

To normalize breast cancer immunohistochemistry (IHC) images, a multi-step method is proposed. Initially, both source and target stained images undergo contrast stretching or STRESS processing, which can be performed using software like GNU Image Manipulation Program. Following this, the images are converted from the RGB color space to the optical density (OD) space using Beer–Lambert’s law. The stained images are then separated into stain vector and stain absorption matrices. The color appearance of the source image is normalized to match the target image using Structure-Preserving Color Normalization (SPCN). The resulting image from the SPCN block is converted back from OD space to RGB space. Finally, the pixels of the contrast-stretched source image are classified using the Robust Self-Sparse Fuzzy Clustering Algorithm (RSSFCA) to identify background and nuclei, thereby determining their locations. This comprehensive approach aims to standardize the color distribution of IHC images, making them more interpretable for pathologists. The schematic representation of the proposed normalization technique is illustrated in Figure 1.

### 2.10. Experiment Settings

In the conducted experiment, we established a series of tests to evaluate five distinct image normalization algorithms, each applied to five different color variations of immunohistochemically stained images, for comparative analysis against our proposed methodology. The algorithms under scrutiny include the color transfer between images method, the histogram specification technique, the Macenko approach, the Structure-Preserving Color Normalization method, and the STRESS technique. Subsequently, we employed QSSIM to assess the degradation in clarity and color saturation of the normalized outputs relative to the original images. The efficacy of histological information preservation is quantified utilizing QSSIM. Furthermore, a three-dimensional histogram visualization of color distribution is employed to facilitate quantitative comparisons. Moreover, we analyze the impact of normalization on classifier performance by utilizing the CNACD to approximately classify breast cancer nuclei based on their central pixels annotated by pathologists. The classification outcomes are derived from six normalization techniques to ascertain whether these methodologies influence the Allred score.

## 3. Results

**Figure 2:**
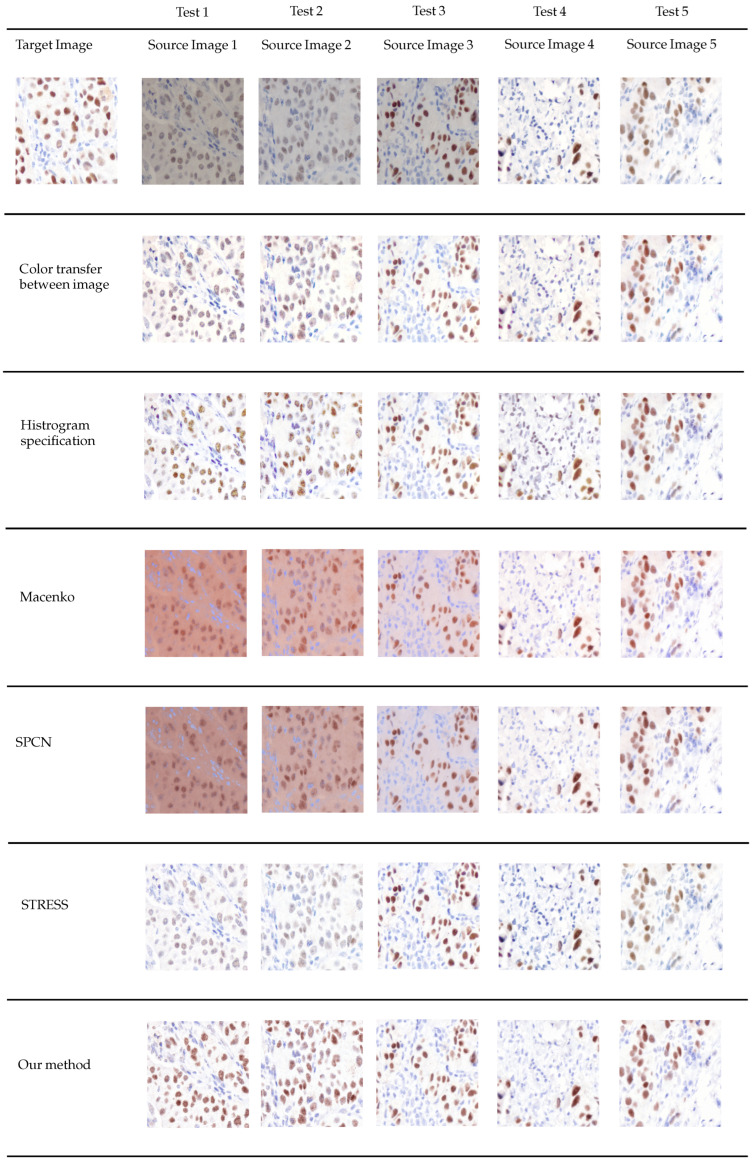
Visual comparison of color normalization methods.

In this study, we executed our model on a computational system equipped with an Intel Core i5 2.3 GHz CPU and 8 GB of RAM. Our proposed normalization methodology was subjected to a comparative analysis against five distinct image normalization algorithms. We conducted tests utilizing five various color modifications of IHC-stained images. The findings are illustrated in Figure 2. The qualitative and quantitative assessments were categorized into three segments. Initially, we assessed the structural similarity of our proposed methodology in relation to the five image normalization algorithms. Subsequently, to demonstrate the efficacy of our normalization technique for Allred scoring, we evaluated the classification accuracy by employing the CNACD classifier. Lastly, for the purpose of quantitative assessment, the 3D histogram visualization of color distribution was utilized and thoroughly analyzed. The outcomes of all methodologies are elaborated upon in the subsequent subsections.

### 3.1. Evaluation of Structure Similarity

For the first evaluation, the QSSIM scores were calcucated using a MATLAB version 2022b script which was written by Kolamen [25]. The detail in the script follows Equation (27). The structure similarity scores for five image normalization algorithms applied with five different color variations of IHC stained images are shown in Table 1.

### 3.2. Classification Performances

To ascertain the classification efficacy, the ground truth was established by the pathologists. The algorithm employed as the classifier was the CNACD methodology. The accuracy (AC) was computed as shown below:

(28) $$AC=\frac{TP+TN}{TP+FN+TN+FP},$$

where TP denotes the aggregate count of true positive cancer nuclei identified within the immunohistochemistry (IHC) image, TN represents the cumulative total of true negative cancer nuclei, FN indicates the overall number of false negative cancer nuclei, and FP signifies the complete tally of false positive cancer nuclei. The accuracy metrics derived from the various normalization algorithms are presented in Table 2.

### 3.3. Quantitative Comparison with 3D Histogram Visualization of Color Distribution

**Figure 3:**
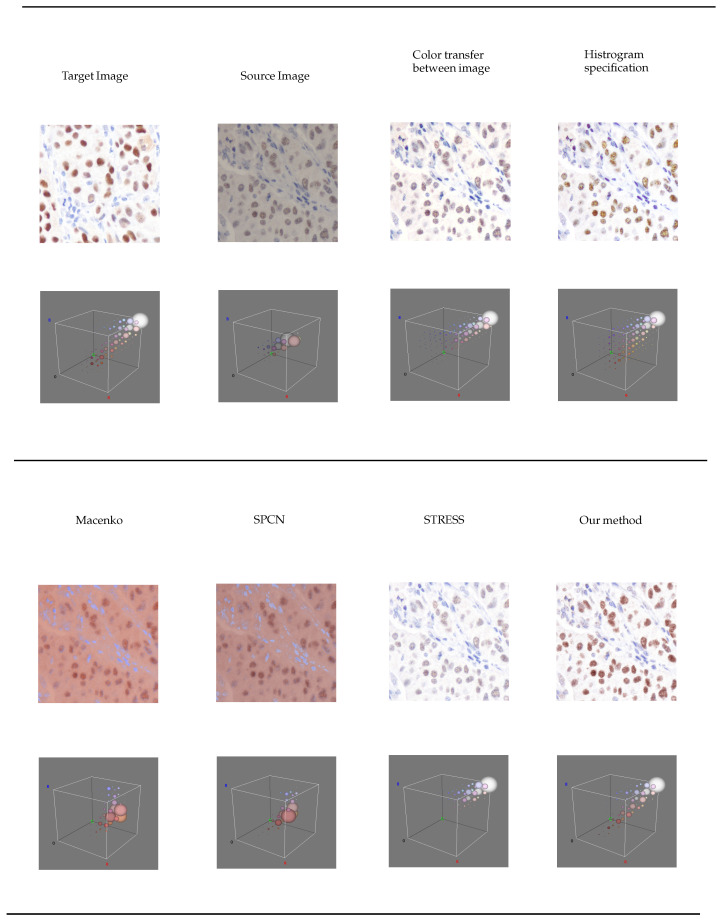
A 3D histogram visualization of color distribution of each color normalization technique.

The three-dimensional histogram representation was generated utilizing the “Color Inspector 3D” version 2.3 [27] plugin within the ImageJ version 1.53 [28] framework. Each color pixel within the image is depicted as the centroid of a circle. Furthermore, the circumference of each circle signifies the prevalence of the corresponding color pixels. This representation is illustrated in Figure 3.

## 4. Discussion

We have conducted a comparative analysis of our proposed methodology against five distinct color normalization techniques: the color transfer methodology as articulated in [26], the histogram specification technique delineated in [2], the Macenko methodology as described in [3], the SPCN technique referenced in [6], and the STRESS methodology elucidated in [19].

Figure 2 illustrates the outcomes of all experimental conditions. It is noteworthy that the backgrounds associated with the results derived from the Macenko and SPCN methodologies do not exhibit a pristine white coloration.

**Figure 4:**
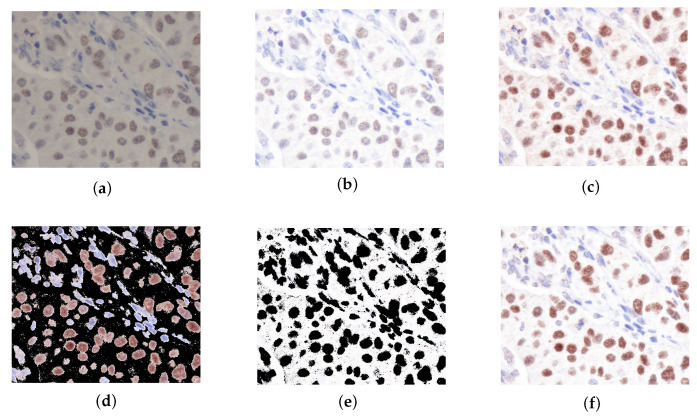
The resultant visual representations derived from each segment of our proposed methodology. (**a**) Original image. (**b**) STRESS original. (**c**) SPCN original. (**d**) Nuclei SPCN original. (**e**) Background STRESS original. (**f**) Normalized original.

Figure 4 shows results from each step of our proposed method. In Table 1, the similarity metrics between the normalized source and the ground truth have been computed employing QSSIM [29]. The findings demonstrate that STRESS consistently achieves superior performance relative to other competing methodologies. According to Figure 4e, thanks to the RSSFCA segmentation error, where certain background regions in the image may be erroneously segmented as nuclei, the tissue structure can be changed, lowering our method’s QSSIM score. Furthermore, the variables for RSSFCA can be optimized through grid search.

In reference to the three-dimensional visualization depicted in Figure 3, the methodologies for color transfer involving image and histogram specification exhibit the presence of purple clusters. Furthermore, it is noteworthy that the color clusters associated with the STRESS methodology are devoid of the brown color cluster. In our proposed approach, the color clusters demonstrate a closer resemblance to those of the target image. However, there are more brownish pixels, but these are not in the nuclei. This reflects the structure changes, consequently, affecting the structure similarity score, QSSIM.

Table 3 delineates the computational complexity associated with each phase of our proposed methodology. The cumulative complexity is denoted as $O(n^{2})$. This complexity is dictated by the term possessing the highest order, specifically STRESS. The STRESS component emerges as the most critical step, as it significantly enhances the image contrast. Furthermore, it is noteworthy that the complexity can be mitigated through the application of the Quantile-Based Retinex (QBRIX) [30] or the Retinex-Based Fast Algorithm (RBFA) [31].

The aggregation of brown-hued regions within the histopathologically stained imagery is of significant relevance as it serves as a basis for the computation of the Allred score. This particular score is instrumental in assessing the therapeutic regimen for breast cancer patients [32].

**Figure 5:**
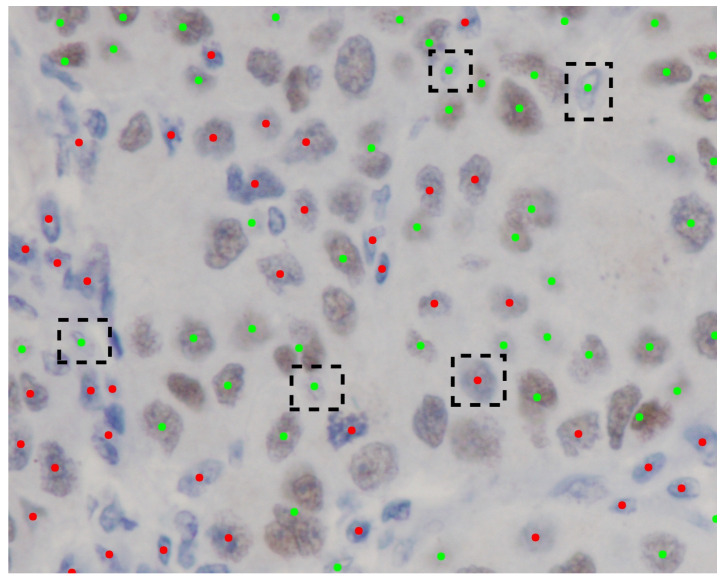
Ground truth Source Image 2 annotated by the pathologists. Green dots signify immunopositive nuclei. Red dots denote immunonegative nuclei. Black dashed rectangles illustrate the erroneous predictions generated by our methodology.

**Figure 6:**
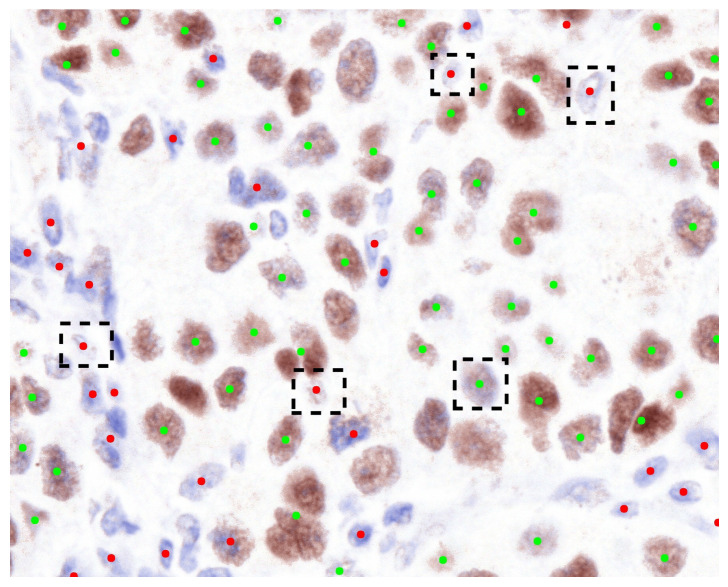
The categorization of nuclei within the normalized Source Image 2 utilizing the methodology we have proposed. Green dots denote immunopositive nuclei, whereas red dots signify immunonegative nuclei. The black dashed rectangles illustrate the instances of erroneous predictions made by our methodology.

Moreover, the results of the classification experiment are presented in Table 2. This table illustrates that the outcomes of our method exhibit a degree of similarity to those of other methods. In Test 1, our method outperforms the performance of other techniques. The classification of the eight true negative nuclei cannot be adequately achieved through the use of a single pixel, as certain brown nuclei, which include blue portions, were identified as immunonegative by the pathologists. Consequently, the classification of these eight nuclei necessitates the involvement of neighboring pixels to enhance the weighting of the classification outcome. Furthermore, the eight nuclei are depicted in Figure 5. The predictive performance concerning the eight nuclei, derived from the normalized output of our method, is illustrated in Figure 6. To evaluate the efficacy of the color features extracted from the normalized images, we have conducted an automated and unsupervised classification of nuclei utilizing Automatic Color Deconvolution (ACD) and determined that it does not substantially influence the accuracy. Although our methodology employs the unsupervised classification technique CNACD, the results yield a performance that is not markedly different from those of other methodologies.

The Allred score is a semi-quantitative method for assessing the estrogen receptor (ER) and progesterone receptor (PR) status in breast cancer IHC slides, yielding a score from 0 to 8 based on positive cell proportion and staining intensity. The assessment is traditionally visual and may introduce subjectivity. Automated methods can be classified as point-based [33] or patch-based [34], with the former focusing on grid points and the latter on image regions, often utilizing deep learning for estimation. Point-based methods exhibit greater precision in low-expression cases, whereas patch-based methods offer efficiency for large Whole Slide Imagings (WSIs) but may miss subtle variations.

The point-based method samples fixed grid points on the image for score estimation. This approach may overlook brown-hued areas. Consequently, the overall score may be compromised. Furthermore, the ACD can improve performance by choosing optimal points rather than relying on grid points. In contrast, the patch-based method necessitates machine learning. Therefore, score accuracy relies on training.

## 5. Conclusions

A novel methodology for color normalization in breast cancer immunohistochemistry (IHC) images is presented in this manuscript. This approach employs sparse stain separation and self-sparse fuzzy clustering, with the objective of more interpretably rendering images for pathologists by standardizing color distribution, thus facilitating more precise Allred scoring for the evaluation of cancer cell proliferation and informing treatment decisions. Notwithstanding its reduced structural preservation in comparison to alternative methodologies, the classification outcomes derived from the point-based approach are analogous to those obtained from other techniques. Furthermore, it has augmented pathologists’ perception of nuclear morphologies and color saturation.

## Figures and Tables

**Table 1:** Quality metrics of various color normalization methods.

	QSSIM				
Color Normalization Method	Test 1	Test 2	Test 3	Test 4	Test 5
Color transfer between image [26]	0.8057	0.8563	**0.9521**	0.9913	0.9815
Histogram specification [2]	0.8132	0.8761	0.9508	0.9827	**0.9918**
Macenko [3]	0.8059	0.8837	0.9227	0.9232	0.9841
SPCN [6]	0.8096	0.8761	0.9157	0.9121	0.9808
STRESS [19]	**0.8870**	**0.9405**	0.9374	**0.9916**	0.9909
Our method	0.7568	0.8166	0.9400	0.9214	0.9823

**Table 2:** The accuracies of nuclei classification by using CNACD.

	Classification Accuracy				
Color Normalization Method	Test 1	Test 2	Test 3	Test 4	Test 5
Original image	82.56	**82.98**	96.10	79.01	73.43
Color transfer between image [26]	88.37	**82.98**	96.10	**80.25**	73.43
Histogram specification [2]	80.23	75.53	96.10	79.01	73.43
Macenko [3]	76.74	79.79	**97.40**	**80.25**	73.43
SPCN approach [6]	74.42	79.79	96.10	**80.25**	73.43
STRESS [19]	88.37	79.79	96.10	**80.25**	**75**
Our method	**90.70**	76.70	96.10	**80.25**	73.43

**Table 3:** Computational Complexity Table (Big-O Analysis) for each step in our proposed pipeline.

Method	Computational Complexity
STRESS [19]	$O(n^{2})$
SS [3]	O(nlogn)
SPCN [6]	O(n)
BLT/IBLT [18]	O(1)
RSSFCA [24]	O(n)
Merge	O(n)

## Data Availability

The original contributions presented in this study are included in the article. Further inquiries can be directed to the corresponding authors.

## Abbreviations

The following abbreviations are used in this manuscript:

IHC	Immunohistochemistry
ACD	Automatic Color Deconvolution
GIMP	GNU Image Manipulation Program
SVD	Singular Value Decomposition
CAP	College of American Pathologists
WHO	World Health Organization
H&E	Hematoxylin and Eosin
DCIS	Ductal Carcinoma In Situ
CD	Color Deconvolution
OD	Optical Density
CS	Contrast Stretching
STRESS	Spatio-Temporal Retinex-like Envelope with Stochastic Sampling
SS	Stain Separation
SPAMS	SPArse Modeling Software
FC	Fuzzy Clustering
RSSFCA	Robust Self-Sparse Fuzzy Clustering Algorithm
SPCN	Structure-Preserving Color Normalization
SNMF	Sparse Non-negative Matrix Factorization
QSSIM	Quaternion Structural SIMilarity
VQM	Visual Quality Matrix
QIP	Quaternion Image Processing
CNACD	Classification of Nuclei in Breast Cancer IHC based on Automatic Color Deconvolution
BLT	Beer–Lambert transformation
IBLT	Inverted Beer–Lambert transformation
TP	True Positive
TN	True Negative
FN	False Negative
FP	False Positive
QBRIX	Quantile-Based Retinex
RBFA	Retinex-Based Fast Algorithm
